# Comprehensive mapping of identical sequences across human proteins emphasizes the widespread issue of shared epitopes in self-antigens

**DOI:** 10.1093/nargab/lqag060

**Published:** 2026-06-22

**Authors:** Guillaume Kellermann, Olivier Croce, Baharia Mograbi, Paul Hofman, Patrick Brest

**Affiliations:** Telomium SAS, Ivry-sur-Seine 94200, France; Université Côte d’Azur, Institute for Research on Cancer and Aging, Nice (IRCAN), Centre national de la recherche scientifique (CNRS), Institut national de la santé et de la recherche médicale (INSERM), Institut Hospitalo-Universitaire (IHU) RespirERA, Fédération Hospitalo-Universitaire (FHU) OncoAge, Nice, France; Université Côte d’Azur, Institute for Research on Cancer and Aging, Nice (IRCAN), Centre national de la recherche scientifique (CNRS), Institut national de la santé et de la recherche médicale (INSERM), Institut Hospitalo-Universitaire (IHU) RespirERA, Fédération Hospitalo-Universitaire (FHU) OncoAge, Nice, France; Université Côte d’Azur, Institute for Research on Cancer and Aging, Nice (IRCAN), Centre national de la recherche scientifique (CNRS), Institut national de la santé et de la recherche médicale (INSERM), Institut Hospitalo-Universitaire (IHU) RespirERA, Fédération Hospitalo-Universitaire (FHU) OncoAge, Nice, France; Laboratory of Clinical and Experimental Pathology, Centre Hospitalier Universitaire (CHU) de Nice, IHU RespirERA, FHU OncoAge, Biobank BB-0033-00025, Nice, France; Université Côte d’Azur, Institute for Research on Cancer and Aging, Nice (IRCAN), Centre national de la recherche scientifique (CNRS), Institut national de la santé et de la recherche médicale (INSERM), Institut Hospitalo-Universitaire (IHU) RespirERA, Fédération Hospitalo-Universitaire (FHU) OncoAge, Nice, France

## Abstract

Shared epitopes pose safety and efficacy issues for T-cell immunotherapy. To characterize the extent of this problem, we performed a computational analysis establishing a complete atlas of shared identical sequences across the human and murine proteomes. Unlike bacterial or viral antigens, self-antigens, including tumor-associated antigens (TAAs), frequently contain sequences of sufficient length to generate identical epitopes in other self-proteins. Epitopes from these shared sequences can theoretically reduce target specificity, confound immunomonitoring studies, and contribute to pre-existing immune tolerance toward TAAs. Notably, a subset of TAAs identified in this atlas is free of this drawback, providing a new criterion for antigen prioritization in cancer immunotherapy. To facilitate the detection of shared sequences, a web server has been made available at https://epitopemapper.ircan.org/ and the open-source code at https://github.com/IRCAN/EpitopeMapper.

## Introduction

T-cell-based immunotherapies, including cancer vaccines and adoptive T-cell receptor (TCR) therapies, rely on the precise recognition of target antigens to achieve therapeutic efficacy while minimizing adverse effects. The success of these approaches depends critically on the specificity of the intended targets while minimizing off-targets. Past efforts to ensure the safety of immunotherapies have mainly been focused on predicting cross-reactivity for different epitopes [1, 2]; however, the obvious problem created by identical sequences shared by different proteins has been neglected [3]. Indeed, two proteins that contain an identical peptide fragment can present an identical epitope to the immune system, possibly leading to off-target autoimmune toxicity. Moreover, the clinical significance of identical shared epitopes extends beyond safety concerns. Preexisting regulatory T-cells recognizing shared epitopes might contribute to explaining the partial tolerance to many tumor-associated antigens (TAA) compared to foreign antigens [4, 5]. Finally, shared epitopes could complicate the interpretation of immunomonitoring studies, where T-cell responses attributed to specific targets may in fact reflect other unintended proteins.

We have recently emphasized the presence of shared epitopes in several cancer vaccines currently being tested in humans [3]; therefore, we realized the need to provide an exhaustive evaluation of this neglected phenomenon for all the antigens of the proteome. To overcome the limitations of current search tools [1, 2], we have developed a computational approach to list all the shared sequences within a proteome. Our analysis of the human proteome reveals striking differences between self and foreign antigens (used in vaccines for infectious disease), identifies TAAs with unique epitope profiles possibly more suitable for immunotherapy, and provides a sequence-level computational atlas of shared sequences that serves as a practical resource for immunologists designing and interpreting T-cell immune responses. The framework developed does not pretend to predict immunogenicity, which remains to be addressed by other methods, but provides an exhaustive sequence-overlap catalog useful to alert for shared sequences in antigens.

## Materials and methods

We developed an open-source suite of Python scripts (EpitopeMapper, KmerProteomeComparator, and EpitopeCrossScanner) to enumerate *k*-mers, compare proteomes and identify shared epitopes. The full code and its full documentation are freely available at https://github.com/IRCAN/EpitopeMapper.

### Proteome data sets

Reviewed human (UP000005640) and mouse (UP000000589) reference proteomes, including all canonical and isoform sequences, were downloaded from UniProt release 2025 _02 (16 April 2025). Human proteome includes 58 520 protein sequences (20 396 canonical + 38 124 isoforms; 32 418 746 amino-acid residues), and mouse proteome includes 41 489 protein sequences (22 568 canonical + 18 921 isoforms; 23 764 281 residues). In addition, sequences of 13 licensed vaccine antigens and of 29 human viral proteomes were retrieved from UniProt on the same date (Supplementary Table S5). For each antigen, the gene-product FASTA file was used as supplied by UniProt.

### *K*-mers enumeration and indexation

*K*-mers were searched using a sliding window of size *k *= 8 (minimal MHC-I) or *k *= 11 (MHC-II 9-mer core + 2 peptide-flanking residues) [6]; every contiguous peptide was extracted with step = 1. *K*-mers containing a masked residue (i.e.: B, J, O, U, X, Z) were excluded.

For each *k*-mer we stored the parent gene symbol, UniProt accession, positional coordinates, and an isoform flag. Splice variants were treated as independent sequences; however, *k*-mers shared exclusively between a canonical sequence and its own isoforms were not classified as shared epitopes.

Definition of the shared epitope index (SEI)

For a gene *g* and *k*-mer length *k*, let

*P_g,k_* = set of distinct *k*-mers encoded by *g*

*P_¬g,k_* = union of *k*-mers encoded by all other genes.


\begin{eqnarray*}
SE{{I}_{g,k}} = 100 \times \frac{{\left| {{{P}_{g,k}} \cap {{P}_{\neg g,k}}} \right|}}{{\left| {{{P}_{g,k}}} \right|}}.
\end{eqnarray*}

SEI-8 and SEI-11 were computed independently for every human and mouse gene. Genes with SEI = 0 were classified as epitope-unique. Intra-protein repeated sequences were retained in all counts without exclusion.

### Cross-species *k*-mer comparison

To quantify conservation of potential epitopes between species, we used the homemade script KmerProteomeComparator, which (i) extracts all k-mers from two proteomes, (ii) partitions them into *shared, only-in-proteome 1*, and *only-in-proteome 2* sets, and (iii) records absolute and relative counts. Pairwise human-versus-mouse analyses were performed for *k* = 8 and *k* = 11.

Detection of shared epitopes between foreign antigens and the human proteome (EpitopeCrossScanner v0.3)

Each vaccine antigen or viral protein was scanned with the same sliding-window algorithm (script EpitopeCrossScanner.py). Identical 8-mer and 11-mer matches in the human proteome were recorded, and SEI values for foreign antigens were calculated analogously to the procedure above.

## Results

### Comprehensive mapping of all shared identical epitopes

Shared epitopes can be identified using NCBI or UNIPROT BLAST. However, these traditional sequence alignment tools, designed to identify homology between full-length proteins, are based on a statistical framework that does not allow an exhaustive identification of all short sequences. PEPMatch was subsequently developed to address this gap by providing 100% recall for short peptide queries [2], but PEPMatch fails to detect shared epitopes. To illustrate this limitation, we submitted a peptide from ASCL2 (HGGASKKL**SKVETLRSAVEYIRALQ**RLLAE) to the PEPMatch online server, but PEPMatch could not detect the embedded 17-aa (underlined) fragment shared with ASCL1 [3], which contains a predicted HLA-DRB1 epitope SKVETLRSAVEYIRA. This demonstrates that PEPMatch does not allow the identification of shared epitopes in the sense addressed by the present work. Furthermore, the PEPMatch server is currently limited to 1000 peptide queries of at most 50 amino acids, making full protein or proteome technically intractable.

In order to perform an exhaustive listing of all possible shared epitopes, we developed Shared EpitopeMapper, a specialized algorithm designed to systematically scan entire proteins and even full proteomes (including all documented splice variants), specifically searching for redundant sequences of 8 and 11 amino acids: https://epitopemapper.ircan.org/. These lengths correspond to the minimal epitopes required for presentation by MHC-I and MHC-II molecules, respectively [6], the cellular arm of adaptive immunity. Although MHC-I typically presents 9-mer peptides and MHC-II 15-mers, shorter sequences are always embedded within longer ones. By using the minimal lengths as our analytical threshold, we ensure that no shared sequences are missed and that all potential minimal epitopes are captured.

A protein sequence then needs to be expressed, processed, presented, and recognized by a TCR to function as genuine MHC epitope. This is not uniformly predictable, owing to tissue-specific differences in proteasomal processing and patient-level genetic variability in HLA alleles. Current HLA binding algorithms fail to predict some epitopes, and very low HLA binding (>10 000 nM) can sometimes lead to detectable immune responses [7, 8], while undetectable T-cell responses toward extremely low-binding epitopes seem physiologically relevant in autoimmunity and cancer immunotherapy [9], warning that classical immunological principles learned from infectious diseases do not apply to predicting or experimentally detecting the epitopes of self-antigens [10]. Furthermore, very low mRNA levels can still lead to detectable MHC-epitope presentation at the cell surface [11] and may have led to unexpected autoimmune damage [12]. Since no universal threshold currently exists to justify excluding a sequence from consideration as a potential functional epitope, at least in some patients, the present atlas intentionally applies no such filter. Accordingly, the conclusions of this resource are independent of any particular HLA allele and apply across the full diversity of the human population. The shared candidate sequences identified here should then be reevaluated in each context-specific condition by second-step analyses incorporating HLA-binding studies, expression data, and proteasomal processing patterns relevant to the specific case under study.

Our systematic analysis of the human proteome generated two complementary datasets that illuminate the complex landscape of sequence redundancy. First, we catalogued for each gene all the other genes that contain identical MHC epitope sequences, whether 8-mers or 11-mers (Supplementary Tables S1 and S2). Second, we approached the problem from the opposite angle by documenting, for each shared epitope sequence, the complete list of genes harboring that particular sequence (Supplementary Tables S3 and S4). This dual gene-centric and epitope-centric framework provides complementary views of sequence overlap.

**Figure 1.**
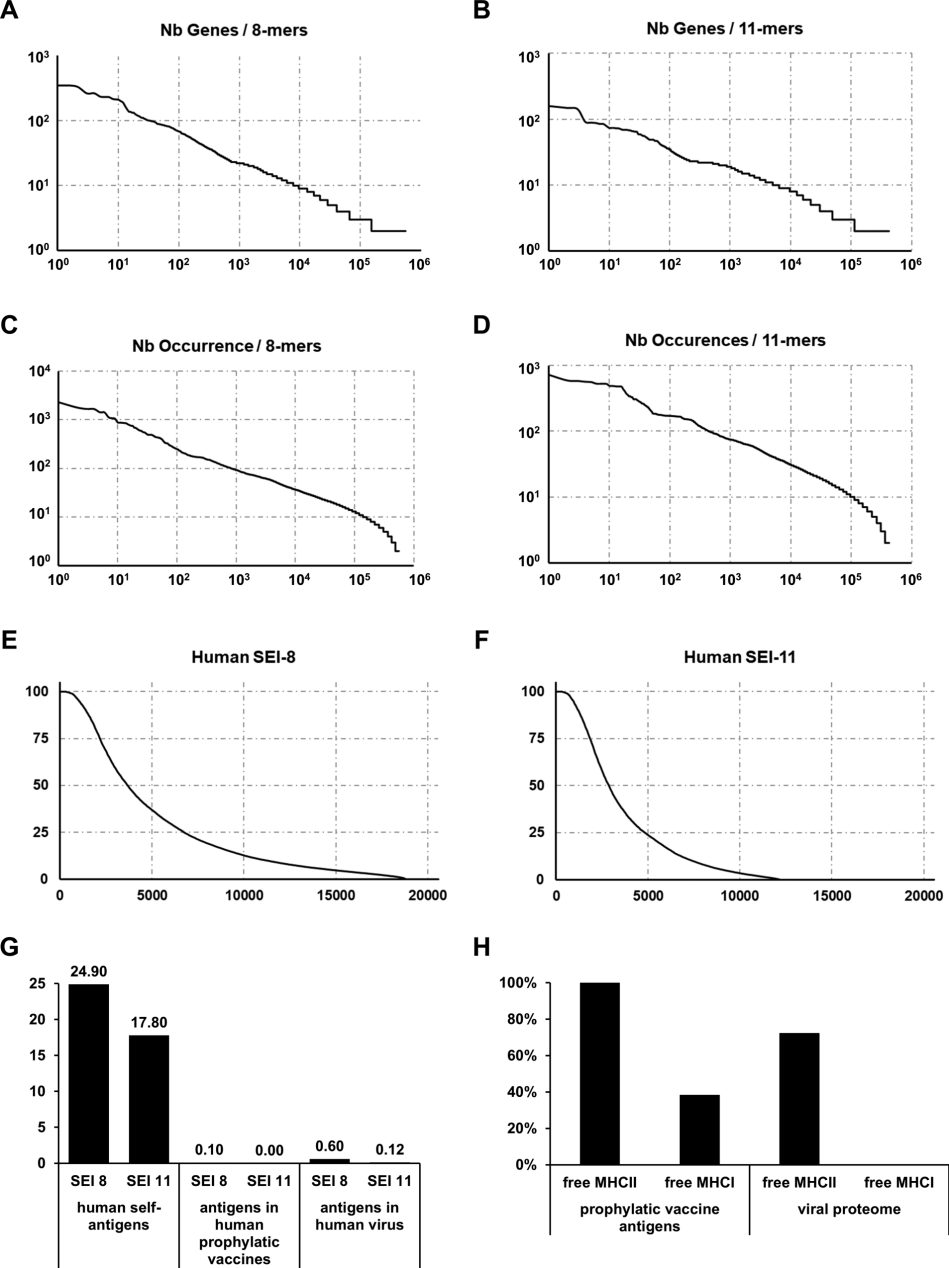
SEI in proteomes. Number of genes sharing at least one identical 8-mer (**A**) or 11-mer (**B**) with other human genes (*x*-axis, log scale), as a function of the number of sharing genes (*y*-axis, log scale). Total proteome-wide occurrence frequency of shared 8-mers (**C**) and 11-mers (**D**). Distribution of SEI-8 (**E**) and SEI-11 (**F**) across all human protein-coding genes. (**G**) Mean SEI-8 and SEI-11 for human self-antigens compared with foreign antigens from prophylactic vaccines and human viral proteomes (Supplementary Table S5). (**H**) Proportion of prophylactic vaccine antigens or viral proteomes free of shared MHC-II or MHC-I epitopes.

The analysis identified 557 930 distinct 8-mer sequences and 421 646 distinct 11-mer sequences that are shared between at least two different proteins. The distribution of sharing frequency shows that the top 100 most widely shared 8-mers each occur in >68 different genes, while even extending to the top 10 000 8-mers reveals sequences shared by at least 9 different genes (Fig. 1A). For longer sequences, the 100 most common 11-mers each appear in over 35 different genes, and the top 10 000 11-mers are each found in at least 8 genes (Fig. 1B).

To account for proteins containing internal repetitive sequences, we calculated the total occurrence frequency of each sequence across the entire proteome. The 100 most abundant 8-mer sequences appear >250 times each throughout the human proteome, while the corresponding 11-mers occur >171 times each (Fig. 1C and D). This analysis captures both inter-protein sharing (sequences found in different proteins) and intra-protein repeats (sequences occurring multiple times within the same protein), thus revealing very complex patterns to consider when trying to predict epitope abundance from gene expression, proteasomal processing, and presentation through the MHC.

### The shared epitope index

To quantify these observations, we introduce the SEI, defined as the percentage of *k*-mers within a protein that are also found in at least another protein of the proteome. We calculated SEI-8 and SEI-11 separately to account for the different minimal requirements of MHC-I and MHC-II presentation pathways (Fig. 1E and F).

The proteome-wide means were 24.9% for SEI-8 and 17.8% for SEI-11, indicating that nearly one-quarter of all potential 8-mer epitopes in human proteins are shared with other proteins. Our analysis also found that 13.9% of all human genes are composed predominantly of shared 11-mer sequences. Moreover, some proteins reached 100% SEI-11, meaning that every possible 11-amino acid segment within these proteins can also be found elsewhere in the proteome.

Conversely, 8.7% of human genes contained no shared 8-mer epitopes, and 41% contained no shared 11-mers (Fig. 1E and F). The gene ontology of the proteins with limited epitope sharing revealed an enrichment for mitochondrial genes, particularly those encoding components of the respiratory chain complexes (Table 1). This observation indicates a lower frequency of orphan genes in nuclear versus mitochondrial genomes.

**Table 1.** Gene ontology of proteins with a high degree of epitope uniqueness

Enrichment FDR	nGenes	Pathway genes	Fold enrichment	Pathways
6.7E-38	850	1830	1.4	Mitochondrion
3.9E-27	291	520	1.7	Mitochondrial matrix
1.2E-24	628	1380	1.4	Organelle envelope
1.2E-24	628	1380	1.4	Envelope
1.5E-23	429	879	1.5	Mitochondrial envelope
3.7E-23	297	559	1.7	Mitochondrial inner membrane
2.9E-22	324	630	1.6	Organelle inner membrane
1.4E-21	198	339	1.8	Mitochondrial protein-containing complex
9.5E-21	399	828	1.5	Mitochondrial membrane
2.6E-15	74	101	2.3	Organellar ribosome

### Antigens used in prophylactic vaccines against infectious disease share little overlap with human proteome

To contextualize the high SEI values of self-antigens, we separately searched for shared epitopes with the human proteome, but now in the antigens used in prophylactic vaccines to prevent infectious diseases. Across 13 foreign antigens from licensed prophylactic vaccines, we identified 18 8-mers that were identical to sequences in the human proteome, representing a total mean of 0.1% SEI-8 and 0% SEI-11. This exceeds the level expected by chance alone, because natural sequences do not use the full space of possibilities [13]. Then, we extended this comparison to the proteome of 29 human viruses and found only a mean of 0.6% for SEI-8 and 0.12% for SEI-11 (Supplementary Table S5). These analyses emphasize a striking difference between foreign and self-antigens (Fig. 1G). *Foreign antigens exhibit minimal sequence overlap with the human proteome, while self-antigens often share a large proportion of epitopes*.

All viral proteomes and some licensed vaccine antigens contained a low content (<2%) of MHC-I epitopes, whereas all vaccine antigens and most viruses are free of shared MHC-II epitopes (Fig. 1H). The shared MHC-II epitopes detected are often limited to low-complexity repetitive sequences in large DNA viruses (Supplementary Fig. S6). Therefore, while a low content of shared MHC-I epitopes is commonly encountered in foreign antigens, shared MHC-II epitopes are much rarer. This might contribute to explaining why CD4 T cells, rather than CD8 T cells, seem to have been evolutionarily selected to play a dominant role in the regulation of immune tolerance. Indeed, CD4 regulatory T cells recognizing self-epitopes can prevent autoimmunity, despite the induction of CD8 T cells [14, 15]. Also, MHC-II shows a stronger and more consistent association with many autoimmune diseases than MHC-I [16].

### Shared MHC-II epitopes are frequent in TAA

**Figure 2.**
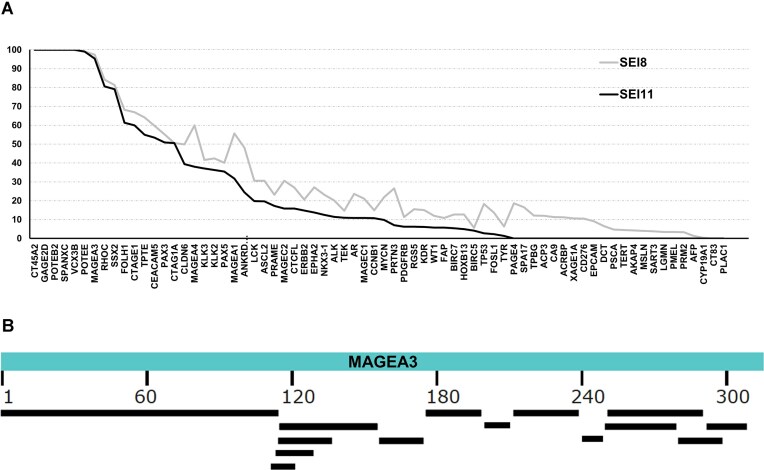
Shared Epitopes in TAA. (**A**) SEI-8 and SEI-11 in common TAA used in therapeutic cancer vaccines. (**B**) Alignments of shared sequences in MAGEA3. The cyan bar represents the full-length MAGE-A3 protein sequence (314 amino acids). The black bars below show the position of the shared identical protein fragments.

In stark contrast to human viruses and prophylactic vaccine antigens, shared MHC-II epitopes are abundant in the majority of the TAAs commonly used in cancer vaccines (Fig. 2A). In some cases, shared MHC-II epitopes accounted for the major portion of the antigen. This extensive sequence overlap may reduce the target specificity of many TAAs and might create off-target issues. The POTEE tumor antigen exemplifies this issue, as it is composed of 99.08% of shared 11-mers. Similarly, >95% of the sequence of MAGE-A3 is covered by partially overlapping fragments from other MAGE proteins. Consequently, the only unique MHC epitopes for MAGE-A3 are restricted to the sequences covering the junctions between the shared sequences and are representing only 4.7% of all the possible 11-mer peptides from this gene (Fig. 2B). These findings demonstrate that shared candidate epitopes must be considered systematically when studying self-antigens, because recent evolution by gene duplication has led to extensive sequence identity across the proteome.

Interestingly, we identified a subset of TAAs that is free of shared MHC-II epitopes and, in some cases, do not contain any shared MHC-I epitopes with the human proteome (Fig. 2A). KK-LC-1, PLAC1, PSCA, PMEL-gp100, CYP19A1, AFP, SART3, CD276, MSLN, AKAP4, TERT, XAGE1, PRM2, LGMN, and ACRBP emerge from this atlas as these antigens exhibit a high degree of sequence uniqueness and provide unambiguous targets for cancer immunotherapies.

### Relevance of mouse models to study shared epitopes

**Figure 3.**
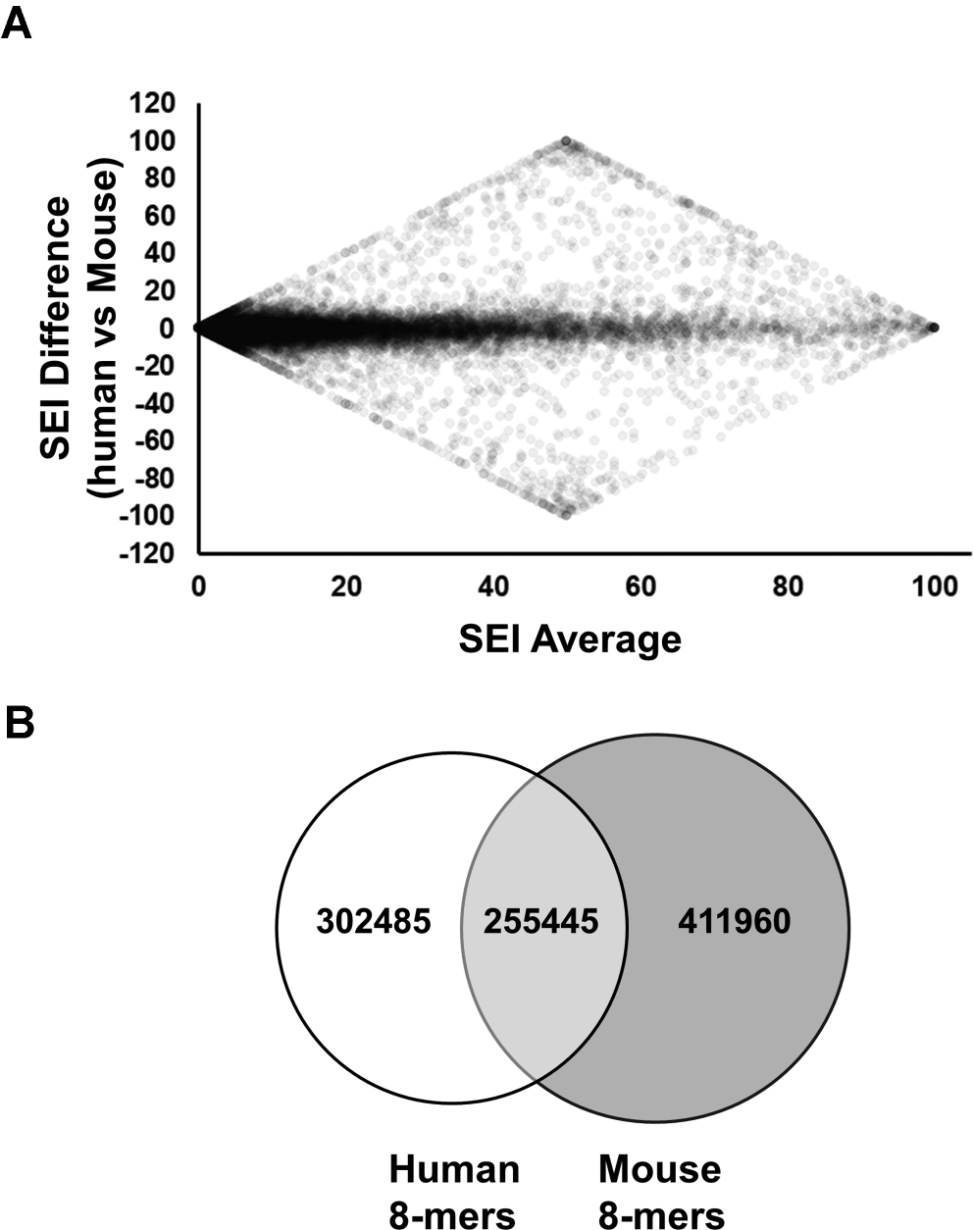
Differential SEI-8 in mice and humans. (**A**) Bland–Altman plot of SEI-8 values across 17 013 orthologous gene pairs; *y*-axis: human-minus-mouse SEI-8 difference; *x*-axis: mean SEI-8. (**B**) Venn diagram of shared 8-mer sequences between the human and mouse proteomes, showing shared and species-specific sequence counts.

By comparing our exhaustive catalog of shared epitopes with mouse orthologs, we found that the SEI of a human gene is usually very close to its corresponding ortholog in mice (Supplementary Tables S6–S9), especially when the SEI is low (Fig. 3A). However, this comparable SEI is mostly due to parallel evolution, as only 41% of shared 8-mer epitopes are identical between both species (Fig. 3B), indicating species-specific differences in the actual epitope sequences despite similar shared patterns. These observations show that mouse models can remain relevant for evaluating the impact of some specific shared epitopes using peptide-based vaccines. However, for protein or mRNA vaccines encoding large antigens, the low conservation of protein sequences substantially limits the translational validity of murine models to assess the safety of such immunotherapies.

## Discussion

This proteome-wide analysis reveals a fundamental asymmetry between foreign antigens used in prophylactic vaccines and self-antigens used in therapeutic cancer vaccines. Prophylactic foreign antigens are free of MHC-II epitopes shared with human proteins, whereas TAAs contain extensive sequences that overlap with endogenous, non-targeted proteins. This high level of epitope sharing can theoretically affect both TAA immunogenicity and tumor specificity, although such functional consequences will need to be confirmed by complementary analyses in each specific case.

Even oncofetal TAAs with restricted tumor expression, such as POTEE [17], may lose their specificity because they share epitopes with other proteins. Pre-existing CD4+ regulatory T cells that recognize these shared epitopes might provide a mechanistic explanation for the partial tolerance often observed to oncofetal TAA [4, 5]. Recognition of the existence of a high SEI in most TAA now calls for a reassessment of the past TAA prioritization rank for cancer immunotherapy [18]. Among the commonly studied TAAs, KK-LC-1, TERT, PLAC1, CYP19A1, AFP, AKAP4, XAGE1, PRM2, and ACRBP possibly emerge as superior candidates due to their high content of unique epitopes and limited somatic expression. These TAAs exhibit greater “foreignness” compared to conventional TAAs and probably also many neoantigens [19]. Alternatively, our comprehensive shared epitope catalog will facilitate the systematic removal of problematic sequences to create reengineered TAAs for the next generation of cancer vaccines, even if only an unknown proportion of the potential epitopes preidentified here will ultimately be proven to be functionally relevant, in the particular context created by each specific case.

The identification of shared epitopes is also critical for an accurate immunomonitoring. For example, the T-cell responses against MAGE-A3₂₁₂₋₂₂₀ after BNT111 vaccination [20] provide an example of this challenge: this epitope is shared with MAGE-A12, a putative off-target associated with previous cases of neurotoxicity [12]. Shared candidate MHC-I sequences must therefore not be overlooked, particularly for applications that bypass CD4+ T cell help, such as TCR-engineered cell therapy.

Shared epitopes may also offer a strategic therapeutic advantage in some settings. Targeting sequences shared across multiple TAAs may broaden therapeutic coverage, reduce vulnerability to antigen-loss escape, and expand patient eligibility beyond criteria based on single-antigen overexpression. When several TAAs collectively present the same shared sequences at differentially elevated levels in tumors, these sequences may represent actionable targets in patients who would be excluded by conventional single-determinant inclusion criteria.

Two limitations of this resource warrant explicit acknowledgment. First, the SEI is a purely sequence-level metric that does not incorporate HLA binding affinity, immunogenicity, antigen processing efficiency, or gene expression data. The *k*-mer sequences reported here are candidates requiring further context-specific validation, not confirmed immunological epitopes. Future extensions integrating multi-omics data for each tissue, tumor type, and stage with pan-HLA-binding predictions could further refine the biological interpretation of the resource. Second, this analysis is restricted to exact sequence identity. It does not address TCR cross-reactivity that can unexpectedly occur despite multiple mismatches [21]. This omission reflects both the current lack of computational reliability in predicting cross-reactivity [22], but also a deliberate conceptual choice: the catalogue of shared epitopes to be excluded from immunotherapy should not be expanded to all the mutated epitopes that could be recognized by a cross-reactive TCR. Adopting such a radical approach would lead to the rejection not only of neoepitope cancer vaccines, but also of all conventional prophylactic vaccines, which is clearly not the conclusion of the present study. Dangerous cross-reactivity remains a major issue for mutated TCRs [21], but it is a lesser concern with native TCRs that have experienced thymic selection.

In conclusion, the gene-centric and sequence-centric catalogs provided here, together with the EpitopeMapper web server and open-source codebase, form an exhaustive sequence-level resource for the systematic identification of all candidate shared MHC epitope sequences across the human and murine proteomes. This resource is intended to support the informed interpretation of immunomonitoring data and the optimization of T-cell-based immunotherapy.

## Supplementary Material

lqag060_Supplemental_Files

## Data Availability

All data are incorporated into the article and its online supplementary material.
