# Supplementary material for: Comprehensive mapping of identical sequences across human proteins emphasizes the widespread issue of shared epitopes in self-antigens
Source: NAR Genom Bioinform. 2026 Jun 22;8(2):lqag060. doi: 10.1093/nargab/lqag060 (PMC13284612; doi:10.1093/nargab/lqag060)
Supplement: lqag060_Supplemental_Files [file lqag060_supplemental_files.zip › +supplementary legends.docx]

**Supplementary Table 1.** List of genes from the human proteome sorted for their content in shared MHC epitopes (11aa).

**Supplementary Table 2.** List of genes from the human proteome sorted for their content in shared MHC epitopes (8aa).

**Supplementary Table 3.** List of shared 11-mers from the human proteome.

**Supplementary Table 4.** List of shared 8-mers from the human proteome.

**Supplementary Table 5.** shared 8-11mers with the human proteome in prophylactic vaccines or viral proteomes.

**Supplementary Table 6.** List of genes from the murine proteome sorted for their content in shared MHC epitopes (11aa).

**Supplementary Table 7.** List of genes from the murine proteome sorted for their content in shared MHC epitopes (8aa).

**Supplementary Table 8.** List of shared 11-mers from the murine proteome.

**Supplementary Table 9.** List of shared 8-mers from the murine proteome.
